# Effects of Kindergarten, Family Environment, and Physical Activity on Children's Physical Fitness

**DOI:** 10.3389/fpubh.2022.904903

**Published:** 2022-06-10

**Authors:** Wenyan Huang, Jiong Luo, Yanmei Chen

**Affiliations:** College of Physical Education, Southwest University, Chongqing, China

**Keywords:** kindergarten environment, family environment, physical activity, healthy physical fitness, mediation, multiple regression

## Abstract

To explore the relationship between kindergarten environmental factors, children's physical activity, and physical fitness, this study uses the stratified random sampling method to obtain 4,600 children in relevant kindergartens. The questionnaire survey and children's physical fitness test were completed with the help of parents and kindergarten staff. The exploratory (EFA) and confirmatory (CFA) factor analysis is used to process the obtained database and set the significance level of all indicators α = 0.05. The results show that kindergarten environmental factors significantly affect children's physical activity and healthy physical fitness. Children with large play areas in these kindergartens, more sports equipment items, who participate in more than three games per week, of no <40 min of each class, with an appropriate number of classes, and excellent teachers' teaching ability have better physical fitness. Family environmental factors significantly affect children's physical activity and fitness. Children with more family sports equipment items, more peers living nearby, safer playing places, more hands and feet, and parents who are good at sports have better performance in health fitness. Children's physical activity not only directly affects their performance of physical fitness, but also plays a dual intermediary role between kindergarten environment and physical fitness, family environment, and healthy physical fitness.

## Introduction

Early childhood is a relatively stable and rapid development stage, and is the primary stage of future skill development. If we can stimulate the development of children's physical function, it will help to improve their physical activity efficiency (1, 2). Early childhood is also a critical period to learn basic motor skills. Maturity alone cannot be fully developed, but the relevant goals can be achieved through the interaction between individual children and the environment (3). The first environment children are exposed to after birth is the family one. Relevant research shows that family housing form (4–6), socioeconomic status (5, 7), birth order of children (8), education level of children's parents (9), and parents' participation in sports associations (10) can provide important information about the impact of the family environment on children's health and physical development. However, due to the limitation of human resources, scholars have not comprehensively discussed these variables. Scholars tend to focus on three factors: the impact of family sports experience (such as outdoor sports games, running and jumping sports game frequency, etc.) on children's physical fitness (11, 12); the impact of the family physical environment (such as safe sports and game places near the family and the amount of sports equipment in the family) on children's health and physical fitness (13, 14); and the impact of family psychosocial environment (such as the number of siblings, birth order, the number of playmates of the same age, and parents' expectations) on children's health and physical fitness (15–17).

On the other hand, with the rapid development of the economy, the proportion of children from two-wage families is increasing. The enrollment rate of 5-year-old children is almost 100%, and more than half of them are sent to Kindergarten at the age of two (18, 19). Kindergarten is an ideal environment for children to enter learning and grow up in group life formally. It plays a vital role in childhood. From the perspective of dynamic physical education, if children can develop good exercise habits in preschool education, this will impact positively on subsequent healthy growth. Relevant research reports show that kindergarten environmental factors (such as space size, venue equipment, sports games, class methods, etc.) also play an essential role in the development of children's health and physical fitness. The survey found that the space available for sports games in kindergartens in China is generally small, and children's activity space is insufficient (20). Children's sports and game courses are inadequate. The opening rate of weekly physical fitness courses and 1-h outdoor activity courses every day is significantly lower than in Japan. The number of sports and game courses is too large, and teachers waste more time maintaining classroom order, which reduces the teaching quality of sports and game courses (21–23). There are also research reports that show the development of children's health and physical fitness is closely related to the kindergarten environment, the total area of the kindergarten, the amount of sports equipment and equipment items, whether the curriculum content is based on games, and whether children have an annual physical fitness test (24–28).

At present, the lack of physical activity in young children has become a common problem worldwide. In the newly issued guidelines for young children's sports, preschool children must accumulate more than 180 min of various types of physical activities throughout the day, including no <60 min of physical activities of medium and above intensity (29). It can be seen that to meet the requirements of the guide, the impact of family and kindergarten environment on children's physical activity cannot be underestimated. Firstly, children's physiological and cognitive characteristics determine that they need their parents to provide sports experience and information, and sports itself is highly infectious. Children's sports socialization process is affected by parents' sports attitudes. Compared with children without support, children with parental encouragement and support are more likely to participate in various sports games and believe that they have more robust sports performance (30, 31). Secondly, children spend most of their time in kindergartens. Whether the teaching of children's physical education curriculum meets the needs of children, especially whether children can reach the expected level of physical activity every day, is quite sensitive to the impact on the development of their basic motor skills. If children do not participate in sports games for enough time, sedentary time will increase, which is bound to affect their health, physical fitness, and movement development level. In fact, at present, there is a severe shortage of physical activity in children in China. The obesity rate and poor vision detection rate of children aged 3– gradually increase with age (31); the uncoordinated proportion of children's movement development is as high as 42 and 67.3% of children cannot master all kinds of health and physical fitness well (32).

To sum up, children's health and fitness are affected by the kindergarten and family environment, as well as the amount of daily physical activity. Among them, kindergarten curriculum content, teaching methods, class atmosphere, situational arrangement, and learning evaluation all affect health and fitness in a flexible manner. Family sports experience, the physical environment inside and outside the family, and psychosocial environment are more complex and changeable. These multi-level interactive and mixed environmental factors affect the development of children's physical fitness (33). Therefore, it is an important research topic to explore the impact of kindergarten and family environmental factors on children's physical activity and physical fitness, and reveal the causal relationship and influence weight between these variables, so as to cultivate children's good exercise habits. At present, the research of foreign scholars mostly focuses on the investigation of the implementation of sports game curriculum. However, some foreign scholars have extended the detection field to the empirical study of the impact of environmental factors on children's health and physical fitness, the overall research on the relationship between various variables is insufficient. Because of this, this study attempts to explore the causal relationship between kindergarten and family environment, children's physical activity, and healthy physical fitness using quantitative research. It boldly puts forward that physical activity may mediate environmental factors and healthy physical fitness, to provide a more reliable practical basis for promoting the development of children's motor skills and motor performance.

## Objects and Methods

### Respondents

This study selects the parents of children in all kindergartens registered by the Education Bureau of the main metropolitan and non-main metropolitan areas of Chongqing as the survey object. The situation of kindergartens in all districts of Chongqing in 2020 through the preschool education network were sorted, including the main urban areas: Yuzhong District, Jiangbei District, Nan'an District, Yubei District, Beibei District, Jiulongpo District, and Shapingba District. The non-main urban areas included: Yongchuan District, Changshou District, Hechuan District, Jiangjin District, Bishan district, and Jiangjin district. To ensure the representativeness of the sample, this study randomly selected four kindergartens in each section and obtained a total of 52 kindergartens (28 in the main urban area and 24 in the non-main metropolitan area); After the establishment of kindergartens, one kindergarten class aged 4, 5, and 6 was randomly selected from each kindergarten, and a total of 156 classes were obtained (84 in the main urban area and 72 in the nonmain metropolitan area).

### Questionnaire Distribution and Recovery

Before the investigation, the members of the research group communicated with the district education bureaus successively, and then negotiated with the heads of the kindergartens after obtaining authorization. Each kindergarten was entrusted to select the client (principal or director) to submit the survey paper to the corresponding parents of children. In total, 4,600 questionnaires were distributed, 4,386 were recovered, and 99 invalid questionnaires (key information variables were not filled in) were eliminated. Finally, 4,287 valid questionnaires were obtained, including 2,311 boys and 1,976 girls, 3,107 public kindergartens and 1,180 private kindergartens, 2,400 in main metropolitan area, and 1,887 in non-main metropolitan area (see Table 1).

**Table 1:** The relationship between children's physical activity, health fitness, and personal essential background variables (*N* = 4,287).

		**Physical activity**				**Healthy physical fitness**			
		**High activity**	**Moderate activity**	**Low activity**	**test (x^**2**^;P)**	**High fitness**	**Moderate fitness**	**Low fitness**	**test (x^**2**^;P)**
Gender	Male	808 (35.0%)	1,178 (51.0%)	325 (14.1%)	22.32; 0.000	952 (41.2%)	1,016 (44.0%)	343 (14.8%)	33.0; 0.000
	Female	560 (28.3%)	1,129 (57.1%)	287 (14.5%)		664 (33.6%)	882 (44.6%)	430 (21.8%)	
	Total	1,368 (31.9%)	2,307 (53.8%)	612 (14.3%)		1,616 (37.3%)	1,898 (44.3%)	773 (18.0%)	
Kindergarten attribute	Public kindergarten	991 (31.9%)	1,696 (54.6%)	420 (13.5%)	5.79; 0.055	979 (40.8%)	1,022 (42.6%)	399 (16.6%)	23.37; 0.000
	Private kindergarten	377 (31.9%)	611 (51.8%)	192 (16.3%)		637 (33.8%)	876 (46.4%)	374 (19.8%)	
	Total	1,368 (31.9%)	2,307 (53.8%)	612 (14.3%)		1,616 (37.3%)	1,898 (44.3%)	773 (18.0%)	
Kindergarten location	Downtown urban area	960 (40.0%)	1,080 (45.0%)	360 (15.0%)	192.53; 0.000	1,010 (42.1%)	1,092 (45.5%)	298 (12.4%)	125.03; 0.000
	No downtown	408 (21.6%)	1,227 (65.0%)	252 (13.4%)		606 (32.1%)	806 (42.7%)	475 (25.2%)	
	Total	1,368 (31.9%)	2,307 (53.8%)	612 (14.3%)		1,616 (37.3%)	1,898 (44.3%)	773 (18.0%)	

### Research Tools

#### Questionnaire Design

Based on a large number of documents and combined with the needs of this study, a “questionnaire on kindergarten and family environment and physical activity” is formed through preparation, modification, sorting, and analysis, including:

Contents of Kindergarten Environment Survey: (1) kindergarten attribute, subordinate area, and surrounding area; (2) Number of kindergarten staff; (3) The area and total area of the kindergarten playing field; (4) Sports equipment items in kindergartens; (5) Teaching activities in kindergartens (such as the number of sports game courses implemented every week, the main contents of game courses, the time of implementing game courses each time, and the number of classes implementing sports game courses in each class); (6) Teachers who teach children's sports and game courses in kindergartens; and (7) Implementation of a health fitness test in kindergartens.

The contents of the investigation of children's family environment: (1) Residential type; (2) Home sports equipment project; (3) Whether the sports and game places near the home are safe; (4) Number of siblings in the family; (5) Education level of parents; (6) Whether parents are good at sports; and (7) Number of playmates of the same age near the family.

To calculate children's physical activity, the calculation formula of exercise participation used by Fox (34) is adopted: degree of exercise participation = exercise frequency × (average exercise intensity + exercise duration). The greater the value, the higher the degree of exercise participation, and the subjects were divided into high, medium, and low categories according to the baseline of children's physical activity. The calculation method of exercise duration is: A. exercise time ≤ 30 min, B. exercise time is 31–40 min, C. exercise time is 41–50 min; D. The exercise time is 51–60 min, and E. the exercise time is ≥61 min (1–5 points are given from a to e, respectively). The calculation method of exercise intensity is, A) not tired at all, B) not tired, C) a little tired, D) very tired, E) very tired (1–5 points from a to e, respectively). The exercise frequency is calculated based on the number of exercise times in a week: A, ≤ 1 time / week, B, 1–2 times per week, C, 3–4 times per week, D, 5–6 times per week, E, more than six times per week (1–5 points are given from a to e, respectively).

#### Health Fitness Measurement of Young Children

The physical fitness of children aged 4 to 6 is mainly measured by the fifth national physique monitoring index system (children's Edition). The contents include:

(1) Walking balance beam (balance capacity); (2) Standing long jump (explosive force); (3) 15 m obstacle running (coordination and agility); (4) Forward bending in sitting posture (flexibility); (5) Continuous jumping with both feet (muscle endurance); and (6) Six test items, including 20 m running (speed), are designed to evaluate children's health and physical fitness. According to the baseline standard of children's health and physical fitness index, the health and physical fitness were divided into three categories: high, medium, and low. To reduce the error, the survey personnel shall discuss before the test, explain the matters that must be required during the test, and strive to standardize the test. The whole test will be completed from May 1 to June 5, 2020. The six test items were tested twice, and the best results were obtained. The testers were composed of 16 postgraduates majoring in physique and health (divided into four groups). To reduce the possibility of test error, the prediction was made in three kindergartens before the formal test, to obtain the reliability among testers (i.e., consistency among testers).

### Validity and Reliability

In terms of validity analysis, after the pre-test survey volume was finalized, eight experts in this field were hired to evaluate the meaning expression (correctness and need) of the questionnaire, and provide suggestions on adding, deleting, or merging and other suggestions. After the questionnaire was recovered, the topic sentences and questionnaire structure were modified one by one according to the opinions put forward by experts and scholars. The semantics and sentences were clear, smooth, and complete. Finally, the average validity score of experts was more than 90 points. In terms of reliability analysis, the questionnaire was tested by test-retest reliability. The research group randomly selected three kindergartens in Beibei District and tested them by retest at 3 weeks. The test-retest reliability was 0.93 (*P* < 0.05).

### Mathematical Statistics

SPSS 21.0 was used to analyze the data, and crosstabs, correlation, and regression analysis were used to explore the correlation between kindergarten and family environmental factors, children's sports participation, and children's health and physical fitness index, using Amos 21.0 to construct structural equation model and explore the mediating effect of physical activity. Significance level setting of all indicators α= 0.05.

## Results

### Comparison Between Children's Background Information and Their Physical Activity and Fitness

Table 1 shows:

(1) According to the overall situation of the respondents, the proportion of high, medium, and low physical activity was 31.9, 53.8, and 14.3%, respectively. Through crosstabs analysis, it was found that there were significant gender differences in children's physical activity, which was affected by the geographical location of kindergartens (Pearson Chi-Square: x^2^ = 22.32, 192.53, P = 0.00, 0.00). Among them, the physical activity of male children was generally higher than that of female children (high activity accounts for 35.0 vs. 28.3%), and the physical activity of children in central urban was generally higher than that in non-main urban areas (high activity accounts for 40.0 vs. 21.6%).

(2) The overall situation of children's health and physical fitness was that the proportion of high, medium, and low fitness were 37.3, 44.3, and 18.0%, respectively. Further analysis shows that there were significant gender differences in children's health and physical fitness. At the same time, affected by the attributes and geographical location of kindergartens (Pearson Chi-Square: x^2^ = 33.0, 23.37, 125.03, *P* = 0.00, 0.00, 0.00), it shows that the health and physical fitness of male children was generally better than that of female (high fitness ratio 41.2 vs. 33.6%). The health and physical fitness of public kindergartens was usually better than that of private kindergartens (high fitness ratio 40.8 vs. 33.8%). The health and physical fitness of kindergartens in central areas was generally better than those in non-main urban areas (high Fitness accounts for 42.1 vs. 32.1%).

### Correlation Analysis Between Family, Kindergarten Environment, Children's Physical Activity, and Physical Fitness

Table 2 shows:

**Table 2:** Correlation matrix between family and kindergarten environment, children's physical activity, and health fitness indicators.

	**X**	**M**	**S**	**Y_**1**_**	**Y_**2**_**	**Y_**3**_**	**Y_**4**_**	**Y_**5**_**	**Y_**6**_**
X: Kindergarten Environment	1.00								
M: home environment	0.04	1.00							
S: Physical activity	0.38*	0.32*	1.00						
Y_1_: Walking balance beam (s)	0.31*	0.05	0.51**	1.00					
Y_2_: Standing long jump (cm)	0.32*	0.09	0.37*	0.32*	1.00				
Y_3_: Run around obstacles for 15 m (s)	0.07	0.27*	0.48**	0.25*	0.24*	1.00			
Y_4_: Sitting posture forward flexion (cm)	0.10	0.08	0.25*	0.34*	0.29*	0.33*	1.00		
Y_5_: Continuous jump with both feet (cm))	0.32*	0.28*	0.55**	0.41**	0.54**	0.27*	0.45**	1.00	
Y_6_: 20 m running (s)	0.41**	0.37*	0.39*	0.30*	0.21*	0.33*	0.52**	0.41**	1.00

“*”, “**” represent the significant levels of 0.05 and 0.01, respectively.

(1) There is relative independence between the kindergarten environment and the family environment. The correlation coefficient is very weak, but there is a significant correlation between kindergarten environment and family environmental factors and children's physical activity (*r* = 0.38^*^, 0.32^*^); Kindergarten environment was significantly correlated with children's “walking balance beam” (0.31^*^), “standing long jump” (0.32^*^), “double foot continuous jump” (0.32^*^), and “20 m run” (0.41^**^), while the family environment was significantly correlated with children's “15 m obstacle running” (0.27^*^), “double foot continuous jump” (0.28^*^), and 20 m run (0.37^*^).

(2) There is a high correlation between children's physical activity and its six indicators of health and physical fitness, that is, the correlation coefficients between children's physical activity and walking the balance beam, standing long jump, 15 m obstacle running, sitting body flexion, continuous jumping off both feet, and 20 m running are 0.51^**^, 37^*^, 0.48^**^, 0.25^*^, 0.55^**^, and 0.39^*^, respectively. In addition, there is a moderate or high correlation between children's physical fitness indicators (Y_1_-Y_6_), which provides a basis for the construction of subsequent mixed models.

### Analysis of the Impact of Kindergarten and Family Environment and Children's Physical Activity on Their Health and Physical Fitness

To reveal the impact of kindergarten environment, family environment, children's physical activity, and other factors on their health and physical fitness, this study uses multi-classification ordered logistic regression method. The standard model of odds ratio (OR) is:


(1) $$\begin{array}{r} \ln\left(\frac{\pi_{il}+\ldots +\pi_{ij}}{\pi_{i\left(j+1\right)+\ldots \pi_{ij}}}\right)=\alpha_{j}-\left(\beta_{1}X_{i1}+\ldots +\beta_{P}X_{iP}\right) \end{array}$$

(I) Multi classification logistic regression model

The cumulative probability has P_1_ ≤ P_1_ + P_2_ ≤ P_1_ + P_2_ + P_3_ ≤ … ≤ P_1_ + P_2_ +… P_J_ = 1. Therefore, J-1 models can be created for J categories. Each cumulative logistic model is like a general binomial logistic model. If the former J category is merged into one category, it will be merged into another category from (J + 1) to the j.

In this study, according to the baseline standard of children's healthy physical fitness (4 ~ 5 ~ 6 years old), walking balance beam (Y_1_), standing long jump (Y_2_), 15 m obstacle running (Y_3_), sitting posture forward flexion (Y_4_), double foot continuous jumping (Y_5_), and 20 m running (Y_6_) are divided into five grades: excellent, good, medium, pass, and fail. The corresponding probabilities are P_1_, P_2_, P_3_, P_4_, and P_5_ in turn. Obviously, P_1_ + P_2_ + P_3_ + P_4_ + P_5_ = 1. Thus (Y_1_-Y_6_), all obey the ordered distribution of 5 classifications. Selecting 13 indicators such as kindergarten environment, family environment and children's physical activity as the influencing variable group, the following four cumulative logistic models can be obtained.


(2) $$\begin{array}{r} \ln\left(\frac{p_{1}}{p_{2}+p_{3}+p_{4}+p_{5}}\right)=\alpha_{i}-\left(\beta_{1}X_{i1}+\ldots +\beta_{P}X_{i\beta }\right),\;j=1 \end{array}$$

(3) $$\begin{array}{r} \ln\left(\frac{p_{1}+p_{2}}{p_{3}+p_{4}+p_{5}}\right)=\alpha_{i}-\left(\beta_{1}X_{i1}+\ldots +\beta_{P}X_{i\beta }\right),j=2 \end{array}$$

(4) $$\begin{array}{r} \ln\left(\frac{p_{1}+p_{2}p_{3}}{p_{4}+p_{5}}\right)=\alpha_{i}-\left(\beta_{1}X_{i1}+\ldots +\beta_{P}X_{i\beta }\right),\;j=3 \end{array}$$

(5) $$\begin{array}{r} \ln\left(\frac{p_{1}+p_{2}p_{3}+p_{4}}{p_{5}}\right)=\alpha_{i}-\left(\beta_{1}X_{i1}+\ldots +\beta_{P}X_{i\beta }\right),\;j=4 \end{array}$$

(II) Four cummulative logistic regression models

Regression model and obtain odds ratio (OR). Generally, for the reference baseline (OR = 1), if OR > 1, it indicates that the influencing factor is favorable for Y. Otherwise, it is negative.

Table 3 shows: (1) The playground area in kindergartens significantly affects children's Y_2_, Y_3_, and Y_6_. The OR value shows that when the area of playgrounds in kindergartens increases from “61–100 m^2^” to “≥ 101 m^2^”, the number of children who significantly improve their physical fitness indexes of explosive power, sensitive coordination, and speed will increase to 1.29, 1.28 and 1.35 times the original number, respectively. The number of sports equipment items in kindergartens significantly affects children Y_1_ and Y_3_. The OR value shows that when the number of sports equipment items in kindergartens increases from “less than six kinds” to “ 16 kinds and above,” the number of children who improve their balance ability, sensitivity, and coordination ability will increase to 1.36 and 1.29 times of the original number, respectively. The number of weekly play classes in kindergartens significantly affects Y_1_, Y_2_, Y_5_, and Y_6_. The OR value shows that when the number of weekly play classes in kindergartens increases from “2 times/week” to “≥ 3 times/week”, the number of children whose balance ability, explosive power, endurance, and speed can be significantly improved will increase to 1.52, 1.47, 1.42, and 1.67 times of the original number, respectively. The length of each game class in kindergarten significantly affects Y_2_ and Y_6_. The OR value shows that when the time of each game class in kindergarten increases from “ <30min / time” to “≥41min / time”, the number of children whose explosive power and speed increase significantly will increase to 1.25 and 1.87 times of the original number, respectively. The number of play classes per kindergarten significantly affects Y_2_, Y_3_, Y_4_, Y_5_, and Y_6_. The OR value shows that when the number of play classes per kindergarten increases from “1 class/time” to “2 classes/time,” the number of children who can significantly increase their explosive power, sensitive coordination, flexibility, endurance, and speed will increase to 1.31, 1.24, 1.39, 1.29, and 1.41 times of the original number, respectively, However, when the number of game classes in kindergartens increases from “2 classes/time” to ≥ “3 classes/time,” the number of children whose explosive power, sensitivity and coordination, flexibility, endurance, and speed decline significantly will reach 0.74, 0.68, 0.70, 0.81, and 0.64 times of the original number, respectively. The professional ability of game teachers significantly affects Y_1_, Y_2_, Y_5_, and Y_6_. The OR value shows that when the professional ability of game teachers is improved from “good ability” to “very good ability,” the number of children with a significant increase in balance ability, explosive power, endurance, and speed will increase to 1.32, 1.29, 1.31, and 1.59 times the original number, respectively.

**Table 3:** Statistical table of logistic regression analysis of the impact of family and kindergarten environment and children's physical activity on their Healthy physical fitness.

		**Y**_**1**_			**Y**_**2**_			**Y**_**3**_			**Y**_**4**_			**Y**_**5**_			**Y**_**6**_		
		**Balance**			**Explosive**			**Sensitivity and**											
		**ability**			**force**			**coordination**			**flexibility**			**endurance**			**speed**		
		**OR**	**t**	**p**	**OR**	**t**	**p**	**OR**	**t**	**p**	**OR**	**t**	**p**	**OR**	**t**	**p**	**OR**	**t**	**p**
X_1_	≤ 60 m^2^ (baseline)	1.00	/	/	/	/	/	/	/	/	/	/	/	/	/	/	/	/	/
	61–100 m^2^	1.03	0.69		1.09	1.24		1.11	1.89		0.94	0.81		0.93	0.92		1.10	1.55	
	≥101 m^2^	1.07	0.85		**1.29**	3.17	*	**1.28**	3.52	*	1.07	1.33		1.03	1.30		**1.35**	4.09	*
X_2_	≤ 9附 (baseline)	1.00	/	/	/	/	/	/	/	/	/	/	/	/	/	/	/	/	/
	10–15 kinds	**1.21**	2.96	*	0.90	1.26		1.17	1.89		0.97	1.09		1.02	0.92		1.10	0.88	
	≥16 kinds	**1.36**	3.61	*	1.13	1.84		**1.29**	3.17	*	1.10	1.48		1.012	1.08		0.97	1.03	
X_3_	1time/week (baseline)	1.00	/	/	/	/	/	/	/	/	/	/	/	/	/	/	/	/	/
	2times/week	**1.47**	12.47	***	1.06	1.53		1.03	0.96		0.97	0.87		0.90	0.63		**1.43**	9.18	***
	≥3 times/week	**1.52**	13.5	***	**1.47**	15.3	***	1.14	1.83		1.06	1.29		**1.42**	8.91	**	**1.67**	18.2	***
X_4_	≤ 30 min/time (baseline)	1.00	/	/	/	/	/	/	/	/	/	/	/	/	/	/	/	/	/
	31–40 min/time	1.04	1.28		1.09	1.47		0.93	0.85		1.06	1.54		0.89	0.97		1.15	1.89	
	≥41 min/time	1.08	1.39		**1.25**	3.29	*	1.02	1.23		1.15	1.81		1.07	1.52		**1.87**	19.2	***
X_5_	1class/time (baseline)	1.00	/	/	/	/	/	/	/	/	/	/	/	/	/	/	/	/	/
	2classes/time	1.05	1.25		**1.31**	8.75	***	**1.24**	4.26	*	**1.39**	6.25	**	**1.29**	4.12	**	**1.41**	5.09	**
	≥3 classes/time	1.10	1.47		**0.7444**	−9.1	***	**0.68**	−5.09	*	**0.70**	−8.21	**	**0.81**	−5.27	**	**0.64**	−7.01	**
X_6_	General ability (baseline 基比)	1.00	/	/	/	/	/	/	/	/	/	/	/	/	/	/	/	/	/
	Better ability	1.08	0.92		1.13	1.79		0.97	1.22		1.11	1.58		1.10	0.96		1.14	1.77	
	Excellent ability	**1.32**	3.960	*	**1.29**	3.25	*	1.05	1.43		1.09	1.90		**1.31**	3.58	*	**1.59**	9.2	***
M_1_	≤ 5 items (baseline)	1.00	/	/	/	/	/	/	/	/	/	/	/	/	/	/	/	/	/
	6–9 items	1.09	1.57		**1.23**	5.66	*	1.15	2.21		0.97	0.71		**1.33**	6.19	**	**1.27**	402	*
	More ten items	1.122	1.69		**1.37**	6.01	*	**1.52**	12.4	**	1.08	1.11		**1.62**	15.3	***	**1.40**	6.51	**
M_2_	Not very safe (baseline 比)	1.00	/	/	/	/	/	/	/	/	/	/	/	/	/	/	/	/	/
	Very safe	**1.34**	4.56	*	**1.18**	2.77	*	**1.23**	3.25	*	0.94	0.81		**1.24**	3.25	*	**1.20**	3.51	*
M_3_	One person (baseline)	1.00	/	/	/	/	/	/	/	/	/	/	/	/	/	/	/	/	/
	More than two people	**1.26**	4.03	*	1.22	3.17	*	**1.72**	12.4	***	1.06	1.77		**1.39**	5.89	**	**1.52**	5.87	**
M_4_	Not good at (baseline)	1.00	/	/	/	/	/	/	/	/	/	/	/	/	/	/	/	/	/
	Commonly	1.04	1.25		0.98	0.88		**1.29**	7.51	**	1.06	1.49		**1.24**	4.02	*	1.06 1.14	1.15	
	Be good at	1.11	1.34		1.06	1.44		**1.32**	6.04	**	1.13	1.19		**1.30**	4.33	*	1.14	1.66	
M_5_	Not good at (baseline)	1.00	/	/	/	/	/	/	/	/	/	/	/	/	/	/	/	/	/
	Commonly	**1.19**	3.22	*	1.05	1.02		1.04	1.50		0.96	0.87		**1.18**	2.81	*	0.93	0.82	
	Be good at	**1.23**	2.98	*	0.99	0.95		1.07	1.42		1.10	1.45		**1.27**	3.24	*	1.05	1.32	
M_6_	≤ 2 person (baseline)	1.00	/	/	/	/	/	/	/	/	/	/	/	/	/	/	/	/	/
	3–4 person	1.02	1.54		0.94	0.95		**1.32**	5.87	**	0.91	0.97		1.12	1.99		**1.26**	3.78	*
	Five persons and above	1.08	1.26		0.99	1.26		**1.48**	6.99	**	1.01	1.23		**1.29**	3.21	*	**1.32**	4.06	*
S	Physical activity	**1.29**	3.25	*	**1.22**	2.84	*	**1.31**	4.02	*	**1.219**	2.81	*	**1.33**	4.26	*	**1.20**	3.02	*
	R^2^/F/P	0.30/18.29/**			0.37/29.25/**			0.41/34.17/**			0.17/4.89/*			0.48/87.89/***			0.51/97.12/***		

*X1, Kindergarten play area; X2, Kindergarten sports equipment project; X3, Number of game classes per week in kindergarten; X4, The kindergarten arranges time for each game class; X5, Kindergarten class level of each game class; X6, Professional ability of game teachers; M1, Home sports equipment project; M2, Safety of playground near home; M3, Number of different siblings; M4, Father is good at sports and ability; M5, Mother's good at sports and ability; M6, Number of playmates of the same age near the family; S, Children's physical activity; Y1, Walking balance beam (s); Y2, Standing long jump (cm); Y3, Run around obstacles for 15 m(s); Y4, Sitting posture forward flexion (cm); Y5, Continuous jumping with both feet (cm); Y6, 20 m running(s)*.

* and ** represent significant levels of 0.05 and 0.01 respectively.

(2) Family sports equipment items significantly affect children's Y_2_, Y_3_, Y_5_, and Y_6_. The OR value shows that when the amount of family sports equipment items increases from “6-9 items” to “10 and above,” the number of children who can significantly improve their explosive power, sensitivity coordination, endurance, and speed will increase to 1.37, 1.52, 1.62, and 1.40 times the original number, respectively; The safety of sports and game venues near the home significantly affects children Y_1_, Y_2_, Y_3_, Y_5_, and Y_6_. The OR value shows that when the safety of sports and game venues near the home changes from “not very safe” to “very safe,” the number of children whose balance ability, explosive power, sensitivity, coordination, endurance, and speed increase significantly will increase to 1.34, 1.18, 1.23, 1.24, and 1.20 times of the original number, respectively. The number of siblings in the family substantially affects children Y_1_, Y_3_, Y_5_, and Y_6_. The OR value shows that when the number of siblings increases from “one person” to “two or more,” the number of children with significant increases in balance ability, sensitivity, coordination, endurance, and speed will increase to 1.26, 1.72, 1.39, and 1.52 times the original number, respectively. The father's sports proficiency and ability significantly affect children Y_3_ and Y_5_. When the father's sports proficiency and ability are improved from “general ability” to “very good,” the number of children's sensitive coordination and endurance will increase significantly to 1.32 and 1.30 times the original number, respectively. Mother's Sports proficiency and ability significantly affect children Y_1_ and Y_5_. When mother's Sports proficiency and ability are improved from “general level” to “very good,” the number of children whose balance and endurance are significantly improved will increase to 1.23 and 1.27 times the original number, respectively. The number of playmates of the same age near the family significantly affects children Y_3_, Y_5_, and Y_6_. When the number of playmates of the same age near the family increases from “ ≤ 3 person” to “≥5 person,” the number of children with a significant increase in sensitivity coordination, endurance, and speed will increase to 1.48, 1.29, and 1.32 times the original number, respectively.

(3) Children's physical activity significantly affects their health and physical fitness in six aspects (balance ability, explosive force, sensitivity and coordination, flexibility, endurance, and speed). When children's physical activity increases by a unit value, the number of children whose balance ability, explosive force, sensitivity coordination, flexibility, endurance, and speed increase significantly will increase to 1.29, 1.22, 1.31, 1.21, 1.33, and 1.20 times the original number, respectively.

### Causal Relationship Between Environmental Factors, Physical Activity, and Children's Health and Physical Fitness

**Figure 1:**
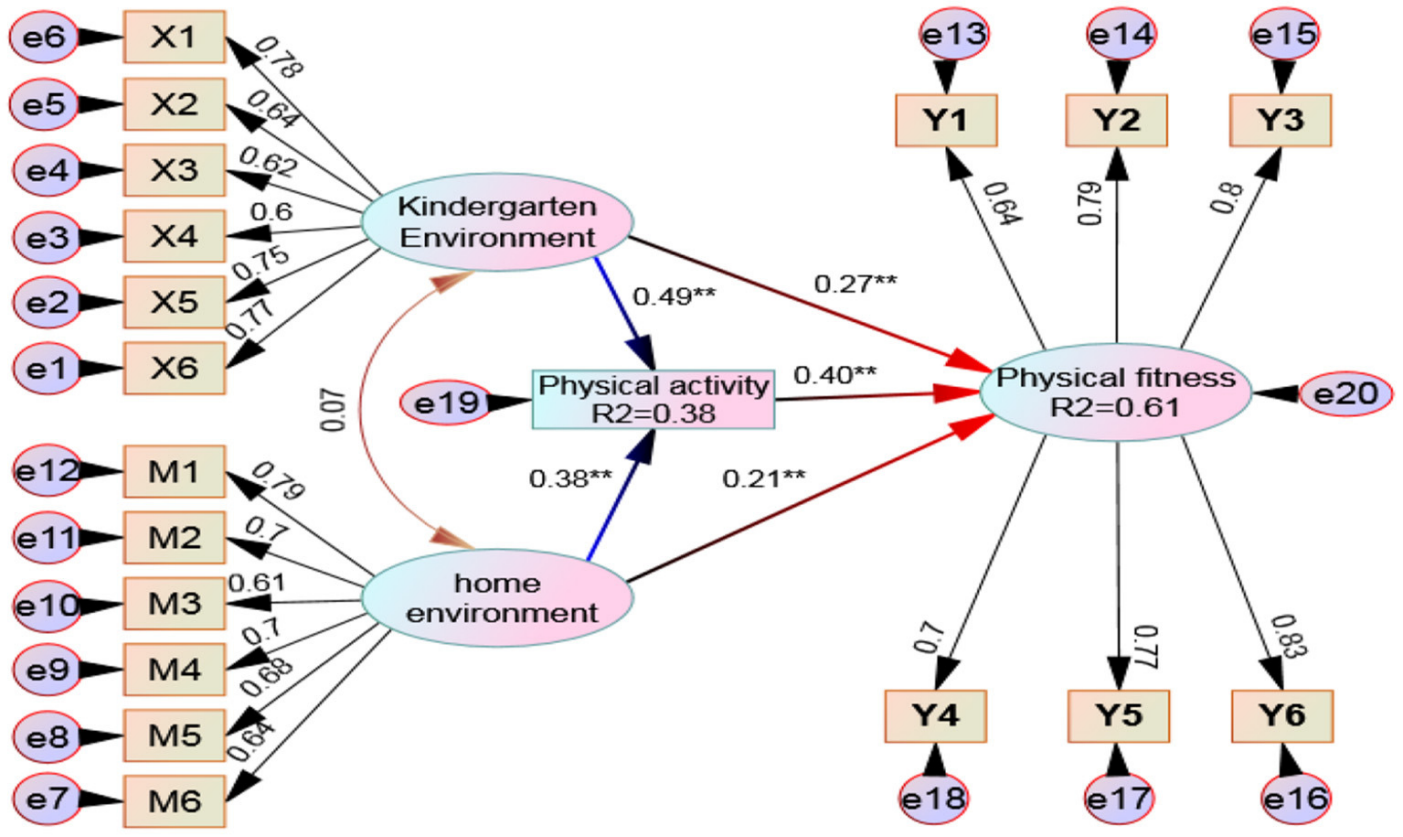
Relationship model between environmental factors, physical activity and children's health and physical fitness. ** means that the path coefficient has reached a very significant level.

Figure 1 is a hypothetical model about the causal relationship between kindergarten and family environment, physical activity, and children's physical fitness. After verification, the model shows:

(1) From the results of absolute fit test: the absolute fit index x^2^ / DF of the model is 2.77, and the corresponding probability *p* = 0.26 < 0.05, indicating that the covariance matrix of the hypothetical model is very suitable for the observed data. The AGFI value of the model is 0.94 (> 0.90 is the adaptation), and the RMSEA is 0.048 (generally, RMSEA < 0.05 is excellent, and 0.05–0.08 is good). From the value-added adaptation test results, IFI = 0.93 (>0.90 is the adaptation), CFI = 0.97 (>0.90 is the adaptation), and TLI = 0.94 (>0.90 is the adaptation). In short, whether absolute fit or value-added fit test, the hypothetical model of this study has a better fit with the actual data.

(2) The mediating effect of physical activity was discussed and calculated according to bootstrap method (34). In this study, non-parametric percentile bootstrap was used to test the significance of the mediating effect. The original data were sampled 2,000 times and the 95% confidence interval (CI) was estimated. Firstly, it is judged that the indirect effect does not contain 0 in the 95% confidence interval and reaches a significant level, indicating that there is an intermediary effect. At this time, if the direct effect contains 0 in the 95% confidence interval, it means that the direct effect is not significant and is a complete intermediary effect; If the indirect effect and direct effect do not include 0 in the 95% confidence interval, both reach a significant level, and if the total effect does not include 0 in the 95% confidence interval, reaching a significant level, it is a partial intermediary effect.

Through Figure 1 and Table 4, it can be seen that the indirect effect of the kindergarten environment on children's physical fitness is 0.19^*^, which is very significant. The confidence interval of 0.087–0.364 obviously does not contain zero, while the direct effect of kindergarten environment on children's physical fitness is 0.27^*^, which is very significant. The confidence interval of 0.106–0.412 obviously does not contain zero. At the same time, the total effect is 0.46^*^, and the confidence interval of 0.156–0.574 also does not contain zero. This fully affirms that physical activity plays a partial intermediary role between the kindergarten environment and children's physical fitness. Similarly, the indirect effect of family environment on children's physical fitness is 0.15^*^, which is very significant. The confidence interval of 0.006–0.287 obviously does not contain zero, while the direct effect of family environment on children's physical fitness is 0.21^*^, which is very significant. The confidence interval of 0.106–0.412 obviously does not contain zero. At the same time, the total effect is 0.36^*^, and the confidence interval of 0.146–0.502 also does not contain zero, Therefore, it can also be judged that physical activity plays a partial intermediary role between children's family environment and children's physical fitness.

**Table 4:** Analysis of the mediating effect of physical activity on the relationship between environmental factors and health and physical fitness.

**Intermediary model I: Kindergarten Environment** ** → Physical**			**Intermediary model II: home environment** ** → Physical**		
**activity** ** → Healthy physical fitness**			**activity** ** → Healthy physical fitness**		
	**Standardization**	**95% confidence**		**Standardization**	**95% confidence**
	**coefficient**	**interval**		**coefficient**	**interval**
Indirect effect: Kindergarten Environment → Physical activity → Healthy physical fitness	0.19*	0.087–0.364	Indirect effect: home environment → Physical activity → Healthy physical fitness	0.15*	0.006–0.287
Direct effect: Kindergarten Environment → Healthy physical fitness	0.27*	0.106–0.412	Direct effect: home environment → Healthy physical fitness	0.21*	0.109–0.368
Total effect: Kindergarten Environment → Healthy physical fitness	0.46*	0.156–0.574	Total effect: home environment → Healthy physical fitness	0.36*	0.146–0.502

*Direct effect, indirect effect and total effect are directly derived from Amos*.

(3) It can be further found from Figure 1 that the judgment coefficient *R*^2^ of kindergarten environment and family environment on physical activity is 0.38, which shows that 38% of the variation of children's physical activity can be caused by the kindergarten environment and family environment, in which the role of family environment accounts for 14% (from the standardized path coefficient *r* = 0.38^*^, 0.38 × 0.38 = 0.14), while the kindergarten environment accounts for 24% (from the standardized path coefficient *r* = 0.49^*^, 0.49 × 0.49 = 0.24). Obviously, the influence of kindergarten environmental factors on physical activity is much higher than that of the family environment. The judgment coefficient *R*^2^ of kindergarten environment, family environment, and physical activity on children's health and physical fitness is 0.61 (7+4+16+19+15%), which shows that 61% of the variation of children's physical fitness can be caused by five aspects. Among the five influences, the direct explanatory power of kindergarten environment, family environment, and children's physical activity on health fitness was 7% (0.27 × 0.27 = 0.07), 4% (0.21 × 0.21 = 0.04), and 16% (0.40 × 0.40 = 0.16) respectively, while the amount of physical activity bears the dual intermediary force, in which the influence of intermediary I is 19% and that of intermediary II is 15%. It can be seen that the amount of physical activity of young children (1 direct influence + 2 intermediary forces = 0.16 + 0.19 + 0.15 = 50%) plays a decisive role in their health and physical fitness.

## Discussion

### Impact of Kindergarten Environment on Children's Health and Physical Fitness

This study found that the amount of sports equipment, the area of game venues, the number of game classes per week, and the number of classes per game class in kindergartens were all related to children's health and physical fitness. From the perspective of the area of kindergarten play space, when the area increases from <60 m^2^ to more than 101 m^2^, children's physical fitness indicators such as explosive power, sensitivity, coordination, and speed are significantly improved. Nathan et al. (35) found that, when the kindergarten game space is relatively reduced and the number of group games decreases, children's aggressive behavior shows a significant increase. Although the research results do not demonstrate that the kindergarten game space area has a positive effect on improving children's health and physical fitness, they do prove the importance of the kindergarten game space area. This study found that when the number of sports equipment items in kindergartens increased from less than ten categories to more than 16 categories, children's health and physical fitness, such as balance ability, sensitivity, and coordination, were significantly improved. This result has been supported by relevant studies (32, 36). The interview further confirmed that the diversity and convenience of sports equipment in kindergartens (such as floor mats, balance beams, large-scale climbing, and other equipment) could not only provide more training opportunities for children's body movements, but also contribute to the improvement of children's health and physical fitness. This study found that weekly sports and game classes in kindergartens can significantly affect children's healthy physical fitness. When the number of classes is increased from 2 times/week to more than 3 times/week, children's balance ability, explosive power, endurance, and speed can be significantly improved. In addition, the length of each game class is also very critical. When the course length is increased from <30min/time to ≥41 min/time, children's ability in explosive power and speed is significantly improved. It can be seen that more than three game classes per week and ≥40 min of game time per class are guaranteed, which is beneficial to promote the physical fitness of children. It is also consistent with the “333 plan” advocated by the newly issued children's sports Guide (3 times a week, 30 min each time, and 130 beats/ min). The latest survey shows that (18, 31) the frequency of sports games in kindergartens in China is mostly ≤ 2 times/week, and the time is <30 min / time. This phenomenon must attract the attention of relevant departments.

In addition, from the teaching methods of children's game courses, this study found a “turning point” phenomenon in the impact of the number of classes in each class on children's physical fitness. Compared with the effect of a single class, if two classes were jointly implemented in each Game class, the results showed that children's explosive power, sensitivity and coordination, flexibility, endurance, and speed could be significantly improved. If more than three classes were jointly implemented in each Game class, then the children's explosive power, sensitivity and coordination, flexibility, endurance, and speed decreased significantly. The reason may be that more than three classes are in the same Game class, and the excessive number of children will significantly increase the difficulty of management, which means that more time is spent on maintaining order, and the time spent on game training decreases. Sport injuries increase at the same time. However, the implementation of sports game courses in a single class is not conducive to children's better sports performance, which seems to imply that they have plenty of game activity time. Still, the number of classes is too small or the class size is too small, which is not conducive to improving children's physical fitness. So, how many classes are needed to jointly implement the game class, or how many teachers are needed in each class, which is more conducive to improving children's physical fitness? What is the causal relationship and possible mechanism between these variables? Finally, the professional ability of game teachers is also worth considering. The study found that if the professional ability of kindergarten sports game teachers is improved to a higher level, children's balance ability, explosive power, endurance, and speed can be significantly improved. Because there are few reports on this research at home and abroad, studies such as that by Tong Tiantian pointed out that (37) kindergarten teachers concurrently serve as sports game course instructors, which will increase children's sports injury rate due to a lack of professional sports cognition. Therefore, from a long-term perspective, it is necessary to carry out systematic study and training for preschool teachers, especially in combination with the experience sharing of actual teachers, to improve teachers' confidence and professional ability in sports game teaching.

### Influence of Family Environment on Children's Healthy Physical Fitness

From the impact of family environmental factors, the number of family sports equipment items has a significant positive effect on children's explosive power, sensitivity and coordination, endurance, and speed, which is consistent with the research of Cong et al. (35) on Japanese children. In addition, Kim and Park (37) found that the convenience and diversity of family sports equipment are conducive to increasing children's engagement in sports games and have positive benefits for improving children's physical fitness, which further supports the findings of this study. This study found that the safety of sports and game venues near the family significantly affected children's balance ability, explosive power, sensitivity and coordination, endurance, and speed, which is also consistent with some previous studies (38, 39). It is believed that children living in communities with old and damaged game facilities near the home have a significant increase in their aimless wandering time, frequent inappropriate events, and significantly poor performance of healthy physical fitness.

From the perspective of the internal environmental structure of the family, this study found that when the number of siblings in the family increases by “1 person,” children's balance ability, sensitivity and coordination, endurance, and speed will increase significantly, which is also supported by previous relevant literature; that is, children with more siblings will perform better in health and physical fitness (40). The interview results show that living in a large family with more siblings can create a fun environment, providing more learning and imitation opportunities for each other, which helps to improve children's health and physical fitness. But there are also inconsistent reports. Scholars found that the balance ability of the first child is significantly better than that of the second and third (41). The study also found that the number of playmates of the same age near the family also has a great impact on children's sensitivity, coordination, endurance, and speed. This result is consistent with the research of sughara (42) and Martzog et al. (43). The latter believes that many leisure activities of today's children are occupied by arts or cultural learning arranged by their parents. There are fewer and fewer opportunities to gather more than 3–5 peers to implement games jointly, and children's healthy physical fitness naturally shows a downward trend. Finally, the father's exercise proficiency significantly affects children's sensitivity, coordination, and endurance, while the mother's exercise proficiency significantly affects children's balance ability and endurance. These findings are consistent with the research results of Hoffmann et al. (44). Therefore, some experts questioned (45, 46) whether children's physical activity, health fitness, and health status are affected by hereditary genes. Parents who are good at sports may bring hereditary genes to children. Therefore, how much influence do environmental factors have? The exploration of these problems is very challenging.

### Physical Activity as a Dual Intermediary Between Environmental Factors and Physical Fitness

#### Effects of Kindergarten and Family Environment on Physical Activity

Previous studies believe that habitual physical activity is a critical factor in developing cognitive ability in early childhood, and a sufficient physical activity environment will significantly accelerate the formation of children's displacement motor skills (47–49). The impact of kindergarten and the family environment on children's physical activity has become a hot topic in children's sports activity research (50). This study found that the multiple regression coefficients (judgment coefficient) *R*^2^ of the kindergarten environment and family environment on children's physical activity was 0.38, and the influence of the kindergarten environment on children's physical activity (24%) was significantly greater than that of the family environment (14%).

The influence of the kindergarten environment on children's physical activity was supported by many previous research results. Pate et al. (51) found that the amount of physical activity of children in kindergartens with different characteristics varies greatly, which may be related to the software and hardware facilities and relevant policies and implementation of kindergartens. Zhang et al. (52) found that children in kindergartens with a supportive environment engaged in medium and high-intensity activities significantly more than those in kindergartens with poor supportive environment. Brown et al. (53) pointed out that the outdoor activity environment of kindergartens (open space, fixed game equipment, balls and equipment, toy wheels, drama props) and teachers' participation enthusiasm significantly affect children's physical activity. Schulman et al. compared (54) 285 American schools and found that the larger the kindergarten area, the higher the students' physical activity and the better the performance of fitness. McCurdy et al. found (55) that the amount and intensity of children's physical activities are affected by the venues, venue facilities, green space area, environmental characteristics, and diversity in the campus. Xin Rou (56) found through telemetry that schools with open surface morphology (such as outdoor walkways), playgrounds, and lawn areas account for a relatively high proportion, and the physical activity of students in these schools is increased. However, there is an apparent lack of research on children's game class week frequency, duration, class number, and teachers' Sports proficiency on children's physical activity. Follow- up research should strengthen the discussion in this regard.

Similarly, the effect of the family environment on children's physical activity has also been supported by many previous studies. Suen et al. (57) found that parents' educational background, parents' restrictions on their children's walking or cycling, and parents' rules on their children's sedentary time are negatively correlated with their children's moderate and high-intensity physical activity after class. Family encouragement, social support, and parents' support for children's play were positively associated with children's physical activity on weekends. Jerstad (58) shows that the distance between individual family and leisure facilities significantly affects children's physical activity, and the frequency of leisure facility use decreases sharply with the increase of length. Frank (59) found that there are more leisure and entertainment facilities, parks, amusement parks, shopping malls, and other facilities near the family, and children's regular walking behavior is better. Hobbs et al. (60) found that after controlling for personal background variables, the closer to the park green space or leisure facilities, the higher the chance of visiting and the greater the amount of physical activity. Another study found that (35) the higher the safety of the environment near the home, the higher the frequency of family physical activity participation, and the greater the amount of physical activity. In short, domestic scholars have little research on the impact of family environmental factors on children's physical activity. Foreign scholars pay more attention to teenagers and middle-aged and elder people, especially the effect of different siblings and the number of playmates of the same age near the family on children's physical activity. This research should be strengthened in the future.

#### Dual Mediating Effect of Physical Activity

This study proposed a mixed structure model diagram between the kindergarten and family environment, physical activity, and children's health and physical fitness, which was adopted after two correction rounds. According to the bootstrap method, it is confirmed that the mediating effect of physical activity is established. The study found that the three variables of kindergarten environment, family environment, and physical activity have a very significant impact on children's health and physical fitness. From the perspective of direct influence, the amount of physical activity is the largest (16%), the kindergarten environment is second (7%), and the family environment is the most minor (4%). What is more noteworthy is that the amount of physical activity not only mediates the kindergarten environment and physical fitness (19%), but also mediates the family environment and physical fitness (15%). In other words, the amount of physical activity plays a dual mediating role of 34%, plus the direct influence of 16%, which has a cumulative force of 50%, which shows the impact of children's physical activity on their physical fitness. This finding has also been supported by many previous scholars. There is a dynamic correlation between the amount of physical activity and the development level of children's motor skills. This correlation may be relatively weak in the early stage of individual life, but it will continue to increase with age (61). The amount of physical activity in childhood is an essential determinant of the development of motor skills and the improvement of healthy physical fitness level. The acquisition and accumulation of children's various exploratory sports experiences need a sufficient amount of activity stimulation. At the same time, an adequate amount of physical activity is conducive to stimulating children's willingness and motivation to participate in sports activities, and then has a positive effect on the level of healthy physical fitness (38). Although the research on the relationship between children's physical activity and their physical fitness has been widely concerned and valued by the academic community, the research on the relationship between them has always lacked sufficient longitudinal research support. There are often dynamic changes between children's physical development, organ function improvement, and basic motor skill development, which determines that the relationship between children's daily physical activity and healthy physical fitness (balance, flexibility, speed, endurance, etc.) is also dynamic. Therefore, the research conclusion will show distinct age and gender characteristics. In the future, more longitudinal studies on the impact of children's physical activity on physical fitness are needed to reveal its impact mechanism fully.

## Conclusion

There are significant gender differences in children's physical activity and health fitness. It is significantly affected by the kindergarten environment and family environment, in which the influence of the kindergarten environment is considerably bigger than that of the family environment. Children's physical fitness can be directly affected by the kindergarten environment, family environment, and physical activity, in which the effect of physical activity is the most significant, the kindergarten environment is the second most, and the family environment is the smallest. The dual intermediary role of physical activity is established. It not only mediates the kindergarten environment and physical fitness, but also mediates the family environment and physical fitness. Therefore, the impact of physical activity on children's physical fitness plays a decisive role.

## Data Availability Statement

The original contributions presented in the study are included in the article/supplementary material, further inquiries can be directed to the corresponding author/s.

## Ethics Statement

The studies involving human participants were reviewed and approved by the Ethics Committee of School of Physical Education, Southwest University (Approval No.: swu-ty202105).

## Author Contributions

WH was mainly responsible for the design of the paper, the preparation of the questionnaire, and participated in the writing of the paper. JL was mainly engaged in the distribution of the questionnaire and data processing and analysis. YC provided decision-making and financial support for this study. All authors contributed to the article and approved the submitted version.

## Conflict of Interest

The authors declare that the research was conducted in the absence of any commercial or financial relationships that could be construed as a potential conflict of interest.

## Publisher's Note

All claims expressed in this article are solely those of the authors and do not necessarily represent those of their affiliated organizations, or those of the publisher, the editors and the reviewers. Any product that may be evaluated in this article, or claim that may be made by its manufacturer, is not guaranteed or endorsed by the publisher.
